# Multichannel EEG abnormalities during the first 6 hours in infants with mild hypoxic–ischaemic encephalopathy

**DOI:** 10.1038/s41390-021-01412-x

**Published:** 2021-04-20

**Authors:** Aisling A. Garvey, Andreea M. Pavel, John M. O’Toole, Brian H. Walsh, Irina Korotchikova, Vicki Livingstone, Eugene M. Dempsey, Deirdre M. Murray, Geraldine B. Boylan

**Affiliations:** 1 INFANT Research Centre, Cork, Ireland; 2 Department of Paediatrics and Child Health, University College Cork, Cork, Ireland

## Abstract

**Background:**

Infants with mild HIE are at risk of significant disability at follow-up. In the pre-therapeutic hypothermia (TH) era, electroencephalography (EEG) within 6 hours of birth was most predictive of outcome. This study aims to identify and describe features of early EEG and heart rate variability (HRV) (<6 hours of age) in infants with mild HIE compared to healthy term infants.

**Methods:**

Infants >36 weeks with mild HIE, not undergoing TH, with EEG before 6 hours of age were identified from 4 prospective cohort studies conducted in the Cork University Maternity Services, Ireland (2003–2019). Control infants were taken from a contemporaneous study examining brain activity in healthy term infants. EEGs were qualitatively analysed by two neonatal neurophysiologists and quantitatively assessed using multiple features of amplitude, spectral shape and inter-hemispheric connectivity. Quantitative features of HRV were assessed in both the groups.

**Results:**

Fifty-eight infants with mild HIE and sixteen healthy term infants were included. Seventy-two percent of infants with mild HIE had at least one abnormal EEG feature on qualitative analysis and quantitative EEG analysis revealed significant differences in spectral features between the two groups. HRV analysis did not differentiate between the groups.

**Conclusions:**

Qualitative and quantitative analysis of the EEG before 6 hours of age identified abnormal EEG features in mild HIE, which could aid in the objective identification of cases for future TH trials in mild HIE.

**Impact:**

- Infants with mild HIE currently do not meet selection criteria for TH yet may be at risk of significant disability at follow-up.
- In the pre-TH era, EEG within 6 hours of birth was most predictive of outcome; however, TH has delayed this predictive value.
- 72% of infants with mild HIE had at least one abnormal EEG feature in the first 6 hours on qualitative assessment.
- Quantitative EEG analysis revealed significant differences in spectral features between infants with mild HIE and healthy term infants.
- Quantitative EEG features may aid in the objective identification of cases for future TH trials in mild HIE.

## Introduction

Hypoxic–ischaemic encephalopathy (HIE) accounts for 1–3 per 1000 live births per year^1^ and is the leading cause of acquired brain injury in term infants. It is clinically graded as mild, moderate and severe. Adverse long-term neurodevelopmental outcome is correlated with increasing severity of encephalopathy.^2–5^

Therapeutic hypothermia (TH) has become standard of care for infants with moderate-to-severe HIE.^6^ To be effective, TH must be commenced early, within 6 hours of birth.^7–9^ However, it can often be difficult to differentiate clinically between mild and moderate encephalopathy in this short timeframe.^10,11^ TH is not currently indicated for infants with mild HIE. Previously, these infants were considered to have normal outcomes^2,12,13^ and so were omitted from TH trials due to the perceived low risk of disability. However, more recent studies highlight significant levels of disability at follow-up.^14–17^ A systematic review by Conway et al. found that 25% of infants with mild HIE had poor neurodevelopmental outcome.^18^ Their pattern of disability appears different to those with moderate to severe HIE. Infants with mild HIE have less motor difficulties but have an increased risk of learning disabilities, emotional and behavioural issues, with 35% requiring school and/or behavioural support at 5 years.^15^ This rate is similar to previous cohorts of infants with moderate HIE who were not cooled.^15^

Despite the lack of appropriate evidence, there has been a drift in practice with some centres providing TH to infants clinically categorised as mild HIE.^19–21^ This is fuelled by fear of both misdiagnosis and litigation.^22^ Trials evaluating TH in infants with mild HIE are required but identification of such infants can be challenging clinically. Disagreement exists regarding both the method and timing of assessment.^2,23^ Clinical and electroencephalography (EEG) assessment tools validated for use at 24 hours are no longer appropriate.^6^ Objective parameters are required to aid in early decision-making in the immediate postnatal period.

EEG plays an important role in caring for newborns with HIE, not only in seizure identification but also in prognostication. EEG findings can evolve rapidly in the first 6 hours of life. Healthy term infants should demonstrate mixed-frequency continuous EEG with regular sleep–wake cycling (SWC) from birth and absence of SWC has been associated with poor neurodevelopmental outcome.^24,25^ Very little information exists about the EEG features in mild encephalopathy. In the pre-TH era, amplitude integrated EEG (aEEG) within 6 hours of birth was the most useful tool for predicting outcome in infants with HIE^26^; however, TH has delayed the predictive ability of aEEG to 48 hours of life.^27–29^ A normal continuous EEG in the first 6 hours of life predicts a normal outcome at 2 years of age.^30^

Heart rate variability (HRV) can be assessed from simultaneous electrocardiography (ECG) recordings. It provides a measure of autonomic function by assessing the difference in time between heartbeats, denoted as either the RR (inter-beat) or NN (“normal” inter-beat) interval. HRV between 12 and 48 hours after birth has the ability to differentiate between grades of encephalopathy and correlate with neurodevelopmental outcome at 2 years,^31^ but no study has examined the ability of early HRV to determine grade of encephalopathy or its early use in mild HIE.

The aim of this study is to identify and describe features of early (before 6 hours of age) EEG and HRV in infants with mild HIE compared to a healthy term group.

## Methods

This was a retrospective study of infants with mild HIE and healthy term infants recruited as part of previous prospective studies in Cork, Ireland between 2003 and 2019. Each study was approved by the Cork Research Ethics Committee. From these cohorts, infants who had EEG recordings before 6 hours of age were identified.

### Cohorts

The non-HIE group was from a previous study examining brain activity in healthy term infants recruited between October 2005 and August 2008.^32^ EEG was recorded on the postnatal ward. Inclusion criteria included infants >37 weeks who did not require resuscitation at birth, had normal cord pH values and had an Apgar score of >8 at 5 min. Sixteen infants had EEG recordings before 6 hours of age.

Infants with HIE were recruited as part of the four prospective cohort studies in the Unified Cork Maternity Services, Cork, Ireland between May 2003 and June 2019. From these cohorts, we identified infants with a clinical diagnosis of mild HIE who had EEG recordings commenced before 6 hours of age. Infants with evidence of perinatal asphyxia (defined as one or more of the following; cord or first postnatal pH <7.1; cord or first postnatal base deficit >16; lactate >9 mmol within the first hour of life; Apgar score <5 at 5 min of life; on-going need for resuscitation at 10 min of life (intermittent positive pressure ventilation or intubation)) were assessed for the presence of HIE using a modified Sarnat exam by experienced clinicians in each cohort (Supplementary Table 1). Mild HIE was defined using the criteria set out by Chalak et al. in the PRIME Study^17,33^ and re-affirmed in a recent expert review.^34^ Specifically, if an infant had any (≥1) abnormality in any of the six domains of the modified Sarnat score but did not meet the criteria for moderate or severe encephalopathy (i.e. ≥3 domains that were categorised as either moderate or severe), they were defined as mild HIE. Multichannel EEG was recorded for between 6 and 72 hours. Inclusion and exclusion criteria of the individual studies are outlined in Table 1. The EEG was recorded from frontal, central, temporal and posterior cortical regions.

**Table 1:** Inclusion and exclusion criteria for the 4 cohort studies.

	Cohort 1	Cohort 2	Cohort 3	Cohort 4
Period	May 2003–Dec 2005	May 2009–June 2011	Feb 2013–Aug 2015	Nov 2017–June 2019
Population	Inborn at 1 of the 3 maternity hospitals in the Unified Cork Maternity Services, Cork, Ireland	Inborn at Cork University Maternity Hospital, Cork, Ireland	Inborn at or outborn and transferred to Cork University Maternity Hospital, Cork, Ireland	Inborn at or outborn and transferred to Cork University Maternity Hospital, Cork, Ireland
Inclusion in the primary study^a^	GA ≥ 37 + 0 All grades of encephalopathy to include the presence of: capillary or arterial pH <7.1 OR lactate >7 mmol/L OR Apgar score 5’ <5 AND abnormal neurological examination	GA ≥ 36 + 0 Signs of asphyxia to include one of: (1) Cord pH <7.1, (2) Apgar score 5’ ≤6 (3) The need for intubation or CPR at birth	GA ≥ 36 + 0 Signs of asphyxia to include: (1) Cord pH <7.1 (2) Apgar score 5’ ≤6 (3) The need for intubation or CPR at birth OR infants with requirement of EEG monitoring in the Neonatal Unit	GA ≥ 36 + 0 Infants with one or more of the following: (1) Apgar score 5’ < 5 (2) Postnatal resuscitation >10 min (3) pH <7.1 or base deficit >16 or lactate >9 mmol/L on cord or first postnatal blood sample AND Presence of abnormal neurological findings on modified Sarnat score at 1 hour of age
Total no. of HIE in the primary study	65	40	59	62
Total mild HIE in the primary study	29	24	36	30
No. in the current study (mild HIE and EEG recording <6 hours of life)	9	16	19	14
Definition of mild HIE	≥1 abnormal finding on modified Sarnat Score at 24 hours of life but not meeting criteria for moderate or severe HIE	Worst grade of encephalopathy in the first 24 hours using modified Sarnat Score but not meeting criteria for TH	Worst grade of encephalopathy in the first 24 hours using modified Sarnat Score but not meeting criteria for TH	1 or more abnormal neurological finding on modified Sarnat Score at 1 hour of life but not meeting criteria for TH
Exclusion from pooling		(1) Infants who received TH (*n* = 1)	(1) Infants who received TH (*n* = 4) (2) Other diagnosis than mild HIE	(1) Infants who received TH (*n* = 2)
Main publications from cohorts to date	Murray et al.^57^ Murray et al.^58^ Murray et al.^59^ Murray et al.^30^ Murray et al.^23^ Murray et al.^15^	Walsh et al.^60^ Walsh et al.^61^ Walsh et al.^62^ Walsh et al.^63^ Looney et al.^64^	O’Sullivan et al.^65^ Rennie et al.^66^ O’Sullivan et al.^67^ Finder et al.^16^ Pavel et al.^68^	

*TH* therapeutic hypothermia, *HIE* hypoxic–ischaemic encephalopathy, *GA* gestational age, *CPR* cardiopulmonary resuscitation, *PA* perinatal asphyxia.

^a^Exclusion from primary studies: infants <36 + 0 weeks’ gestation; known genetic disorders and/or inborn errors of metabolism; confirmed sepsis (positive blood or CSF cultures).

### EEG analysis

#### Qualitative

First, all continuous EEG data available before 6 hours of age for the HIE and non-HIE groups were visually assessed, and the background pattern was graded as normal or mildly abnormal background by two neonatal EEG reviewers independently with complete agreement for both grades. As slightly different EEG recording locations were used in the HIE and non-HIE groups, it was not possible to fully blind the reviewers to study group (in the non-HIE group, posterior electrodes were located over the right and left parietal regions rather than occipital regions). Our group has previously developed a standardised grading scheme to analyse EEG features of preterm EEG^35^ and we extended this for the term EEG using seminal works from Lamblin, Andre, d’Allest and others.^36,37^ One EEG reviewer then identified specific qualitative features in both groups according to this assessment scheme.

Qualitative analysis of the EEG was divided into three main categories. Category 1 describes temporal organisation (SWC and features of continuity). Category 2 identifies if abnormal waves are present (immature or deformed waves, sharp waves or diffuse delta waves) and Category 3 describes abnormal features of a term EEG (asymmetry, asynchrony, discontinuity, seizures or low voltage activity). EEGs were reviewed and the presence or absence or the various features were noted.

#### Quantitative

All EEGs were then included in the quantitative analysis. One-hour epochs of each EEG assessed in the qualitative analysis before 6 hours of age were selected to include a full sleep cycle if present and as little artefact as possible. Remaining artefacts were annotated and removed. EEGs were quantitatively assessed using multiple features of amplitude, spectral shape, and inter-hemispheric connectivity using the NEURAL (**N**eonatal **E**eg feat**UR**e set in m**A**t**L**ab) software package (version 0.4.3).^38^

Measures of spectral shape included spectral power, spectral flatness (a measure of spectral entropy) and spectral difference (a measure of difference in spectral shape over time).

As different EEG or aEEG machines use different algorithms to generate an aEEG channel, we use the range-EEG (rEEG) as an alternative. This filtered and time-compressed representative of EEG is similar to the aEEG but has a unique definition, which therefore allows for standard quantitative measures.^38^

#### Heart rate variability

HRV was computed from the same 1-hour epochs used for quantitative EEG analysis in both the HIE and non-HIE groups. *R*-peaks were automatically identified using a HRV software application (HRV Analysis, University College Cork, Cork, Ireland) and then visually inspected and corrected if necessary. Artefacts were annotated and removed. Quantitative HRV features were extracted from the R-R interval^39^ including both time-domain and frequency-domain features. These features are consistent with previous neonatal studies.^31,39^

### Statistical analysis

Statistical analysis was performed using IBM SPSS Statistics (version 24.0, IBM Corp, Armonk, NY, U.S.A.). Continuous variables were described using the mean and standard deviation (SD) or the median and interquartile range (IQR) and categorical variables using frequency and percentage. For comparisons between the two groups (mild HIE, non-HIE), the Mann–Whitney test was used for continuous variables and Fisher’s exact test for categorical variables. Receiver operator characteristic (ROC) curve analysis was used to assess the predictive ability of quantitative EEG and HRV features and qualitative EEG features in identifying mild HIE. All tests were two sided and a *p* value <0.05 was considered statistically significant.

## Results

### Population

#### Non-HIE

Sixteen healthy term infants were included in the non-HIE group. Mean birth weight was 3497 g (SD 381 g) and median gestational age was 39.3 weeks (IQR 38.9–40.6 weeks). Fifty percent (*n* = 8) were born by elective lower section caesarean section (LSCS) and 31% (*n* = 5) were born by spontaneous vaginal delivery (Table 2).

#### Mild HIE

Fifty-eight infants with mild HIE were included. Mean birth weight was 3467 g (SD 527 g) and median gestational age at birth was 40.4 weeks (IQR 39.2–41.3 weeks). Forty-seven percent (*n* = 27) were born by instrumental assisted vaginal delivery and 26% (*n* = 15) were born by emergency LSCS (Table 2).

**Table 2:** Demographics and mode of delivery of the infants included.

	Non-HIE *n* = 16	Mild HIE *n* = 58
Gestational age at birth (weeks) [median (IQR)]	39.3 (38.9–40.6)	40.4 (39.2–41.3)
Birth weight (g) [mean (SD)]	3497 (381)	3467 (527)
Mode of delivery [*n* (%)]		
SVD	5 (31)	14 (24)
Instrumental	1 (6)	27 (47)
Emergency LCSC	2 (13)	15 (26)
Elective LCSC	8 (50)	1 (2)
Not documented	0 (0)	1 (2)

*IQR* interquartile range, *SD* standard deviation, *SVD* spontaneous vaginal delivery, *LCSC* lower section caesarean section.

### Qualitative analysis

Qualitative analysis of the EEG was divided into three main categories as described above: temporal organisation, abnormal waves, and abnormal features. The main results are displayed in Table 3.

**Table 3:** Qualitative EEG features of infants with mild HIE and infants in the non-HIE group.

	Non-HIE *n* = 16 *n* (%)	Mild HIE *n* = 58 *n* (%)	*p* Value*	AUC (95% CI)
Group 1 (temporal organisation)				
Normal sleep–wake cycling	16 (100)	31 (53)	**<0.001**	0.73 (0.67–0.80)
Predominant continuous activity	16 (100)	47 (81)	0.107	0.59 (0.54–0.65)
Group 2 (abnormal waves)				
Diffuse delta waves	0	28 (48)	**<0.001**	0.74 (0.68–0.81)
Sharps (diffuse/excessive/focal/negative)	0	17 (29)	**0.016**	0.65 (0.59–0.71)
Deformed waves	0	6 (10)	0.329	0.55 (0.51–0.59)
Immature waves	0	5 (9)	0.579	0.54 (0.51–0.58)
Mechanical brushes	0	2 (3)	1	0.52 (0.49–0.54)
Positive temporal sharp waves	0	2 (3)	1	0.52 (0.49–0.54)
Positive rolandic sharp waves	0	0		
Group 3 (abnormal features)				
Periods of discontinuity	0	14 (24)	**0.031**	0.62 (0.57–0.68)
Low voltage	0	12 (21)	0.058	0.60 (0.55–0.66)
BIRDs	0	2 (3)	1	0.52 (0.49–0.54)
Asymmetry	0	2 (3)	1	0.52 (0.49–0.54)
Asynchrony	0	1 (2)	1	0.51 (0.49–0.53)
Burst suppression	0	0		
Isoelectric	0	0		
Periodic lateralised epileptiform discharges	0	0		
Seizures	0	0		
Status	0	0		

Statistically significant *p* < 0.05 values are in bold.

*p Value from Fisher’s exact test.

#### Non-HIE group

Infants in the non-HIE group demonstrated normal SWC with continuous mixed-frequency activity with absence of abnormal waves and features.

#### Mild HIE

On assessing temporal organisation, 53% demonstrated clear SWC. Approximately 34% had prolonged inter-burst intervals in quiet sleep. Regarding abnormal waves and features, 48% had diffuse slow waves and 29% had excessive sharp waves. 21% were noted to have low voltage recording and 19% were excessively discontinuous with 24% of the EEGs showing periods of discontinuity (<50% of the recording).

Overall, 72% of the infants with mild HIE had at least one abnormal EEG feature in the first 6 hours of age, including absent or abnormal SWC, intermittent discontinuity, diffuse slow wave activity or excessive sharp waves. The most striking difference visually was the high frequency of slow and sharp waves, periods of excessive discontinuity and lower amplitude. ROC analysis revealed that the absence of SWC or presence of diffuse slow waves were the features that were most predictive of mild HIE.

### Quantitative analysis

Epochs were analysed at a median time of 0.6 hours (IQR 0.3–1.2 hours) after the start of EEG recording. Quantitative analysis revealed significant differences in spectral features between infants with mild HIE and those without (Table 4). 91% of spectral power in the EEGs of infants with mild HIE was in the delta band (<4 Hz), with 95% total power <5 Hz. Both spectral flatness and spectral difference were significantly lower in the delta and theta frequency bands for the mild HIE group compared with the non-HIE group. ROC analysis revealed that these features were also most predictive of mild HIE.

There were no differences between groups in quantitative measures of amplitude (which included rEEG measures), and no differences in measures of inter-hemispheric coherence.

**Table 4:** Quantitative EEG features of infants with mild HIE compared with the non-HIE group.

	Non-HIE [med (IQR)] *n* = 16	Mild HIE [med (IQR)] *n* = 58	*p* Value*	AUC (95% CI)
Amplitude				
Spectral power FB1^a^ (µV^2^)	282.1 (224.9, 386.0)	332.9 (238.0, 637.1)	0.309	0.59 (0.47–0.70)
Spectral power FB2 (µV^2^)	17.5 (15.1, 23.9)	18.5 (13.7, 25.8)	0.940	0.51 (0.39–0.63)
Spectral power FB3 (µV^2^)	9.1 (8.1, 11.6)	8.2 (5.9, 10.8)	0.162	0.62 (0.50–0.73)
Spectral power FB4 (µV^2^)	4.7 (4.1, 5.7)	5.0 (3.5, 8.4)	0.748	0.53 (0.41–0.65)
rEEG median (µV)	52.1 (44.0, 55.1)	47.4 (42.4, 54.9)	0.379	0.57 (0.45–0.69)
rEEG lower margin (µV)	27.0 (22.2, 31.1)	26.1 (22.1, 30.9)	0.667	0.54 (0.41–0.65)
rEEG upper margin (µV)	94.6 (91.3, 97.1)	90.7 (76.7, 103.9)	0.350	0.58 (0.45–0.69)
Spectral shape				
Spectral edge frequency (Hz)	6.3 (5.3, 6.7)	5.2 (3.6, 5.9)	**0.008**	0.72 (0.61–0.82)
Spectral relative power FB1 (%)	89.3 (88.0, 91.1)	91.0 (89.6, 93.0)	**0.023**	0.69 (0.58–0.80)
Spectral relative power FB2 (%)	5.8 (5.2, 6.7)	4.9 (3.7, 5.9)	**0.021**	0.69 (0.58–0.80)
Spectral relative power FB3 (%)	3.0 (2.5, 3.4)	2.2 (1.8, 2.5)	**0.001**	0.77 (0.65–0.86)
Spectral relative power FB4 (%)	1.7 (1.4, 1.9)	1.4 (1.1, 1.8)	0.371	0.42 (0.31–0.55)
Spectral flatness FB1	0.47 (0.41, 0.52)	0.42 (0.29, 0.48)	**0.035**	0.68 (0.57–0.79)
Spectral flatness FB2	0.89 (0.88, 0.90)	0.88 (0.86, 0.89)	**0.007**	0.73 (0.61–0.82)
Spectral flatness FB3	0.89 (0.87, 0.90)	0.89 (0.87, 0.91)	0.440	0.57 (0.44–0.68)
Spectral flatness FB4	0.76 (0.71, 0.77)	0.78 (0.72, 0.82)	0.139	0.63 (0.51–0.74)
Spectral difference FB1	0.010 (0.008, 0.011)	0.007 (0.004, 0.009)	**0.003**	0.75 (0.64–0.85)
Spectral difference FB2	0.026 (0.024, 0.029)	0.023 (0.021, 0.026)	**0.007**	0.73 (0.61–0.82)
Spectral difference FB3	0.021 (0.018, 0.022)	0.020 (0.018, 0.022)	0.875	0.49 (0.36–0.60)
Spectral difference FB4	0.010 (0.009, 0.012)	0.011 (0.009, 0.013)	0.179	0.61 (0.50–0.73)
Inter-hemisphere connectivity				
Coherence FB1	0.172 (0.122, 0.195)	0.118 (0.088, 0.202)	0.379	0.43 (0.31–0.55)
Coherence FB2	0.073 (0.054, 0.084)	0.049 (0.035, 0.084)	0.128	0.37 (0.26–0.49)
Coherence FB3	0.048 (0.044, 0.068)	0.046 (0.035, 0.079)	0.562	0.45 (0.34–0.57)
Coherence FB4	0.036 (0.032, 0.045)	0.038 (0.030, 0.074)	0.647	0.54 (0.41–0.65)

Statistically significant *p* < 0.05 values are in bold.

*p Value from Mann–Whitney test.

^a^FB1 = 0.5–4 Hz; FB2 = 4–7 Hz; FB3 = 7–13 Hz; FB4 = 13–30 Hz.

### Heart rate variability

There were no differences between infants with mild HIE and non-HIE groups (Table 5).

**Table 5:** Quantitative HRV features of infants with mild HIE compared with control population.

Feature	Non-HIE [med (IQR)] *n* = 16	Mild HIE [med (IQR)] *n* = 47	*p* Value*	AUC (95% CI)
Mean NN (msec)	504.6 (477.0–537.6)	490.5 (465.4–520.9)	0.347	0.58 (0.46–0.71)
SDNN (msec)	29.0 (13.9–34.7)	20.8 (15.2–29.2)	0.294	0.59 (0.46–0.71)
VLF power (msec^2^)	4142.5 (1017.3–6295.0)	1888.5 (950.5–3419.6)	0.076	0.65 (0.52–0.77)
LF power (msec^2^)	487.2 (158.1–1051.8)	323.8 (146.8–615.1)	0.227	0.60 (0.47–0.72)
HF power (msec^2^)	8.9 (3.0–32.8)	10.1 (3.5–31.9)	0.906	0.49 (0.36–0.62)
LF/HF ratio	50.2 (26.2–74.4)	34.4 (14.1–65.4)	0.102	0.64 (0.50–0.75)
TINN (msec)	82.0 (58.6–101.6)	54.7 (46.9–76.2)	0.102	0.64 (0.50–0.75)

*SD* standard deviation, *NN* normalised RR interval, *VLF* very low frequency, *LF* low frequency, *HF* high frequency, *TINN* triangular interpolation of the NN interval histogram.

*p Value from Mann–Whitney test.

## Discussion

This is the first detailed study describing multichannel EEG and HRV in infants with mild HIE within 6 hours of birth. We have found significant differences between the EEG features of infants with mild HIE and healthy term infants. Our qualitative analysis identified the presence of specific abnormal EEG features in the HIE group. SWC should be present from birth^40^ and the absence of SWC is associated with poor neurodevelopmental outcome.^24,25^ Good quality sleep is crucial for an infant’s development and studies have shown disruptions in both the presence and composition of the SWC in term infants post asphyxia injury, specifically a decrease in the proportion of active sleep and an increase in the amount of quiet and indeterminate sleep within the sleep cycle.^41–44^ Almost half of the infants with mild HIE had absent or poor SWC in the first 6 hours, active sleep was absent in 35% and quiet sleep was abnormal, as it contained prolonged inter-burst intervals, in 34%. It is important to consider the effect that interventions in the neonatal unit may have on SWC; however, it is our practice to only perform necessary procedures and cares on admission and nurse these infants in incubators with minimal handling thereafter. We have also previously shown that normal continuous SWC activity is present from birth in healthy control infants without perinatal asphyxia.^40^ As well as altered SWC, we also found that 24% of the EEGs in infants with mild HIE had periods of excessive discontinuity and 19% were predominantly discontinuous. Excessive sharp waves were seen in 29%. Qualitative analysis demonstrated excessive slow waves in infants with mild HIE, which was also confirmed on quantitative analysis.

Previous studies have shown altered HRV features with increasing grade of encephalopathy^31,39^; however, no study to date has assessed HRV before 6 hours of age. Animal studies have found an increased variability in the HRV (increased SDNN) in preterm foetal sheep between 4 and 6 hours post occlusion with severe hypoxia–ischaemia compared with those with mild hypoxia–ischaemia or controls.^45^ In this study, we found no differences in measures of HRV in infants with mild HIE compared with healthy term infants in the first 6 hours.

There is a growing body of evidence that infants with mild HIE have significant levels of disability at follow-up, yet no current evidence or guidance exists regarding potential therapeutic interventions in this group. This, coupled with the medico-legal implications of not offering TH to infants who may have benefitted, leads to unease and difficulty for clinicians in objective decision-making regarding treatment. In addition, there is therapeutic creep and many centres are now cooling infants with mild HIE.^19–21^

Although TH has a wide margin of safety, it is not without consequences. Inappropriate TH has a number of potential adverse consequences. Animal studies have suggested that induced hypothermia in a normal brain may lead to apoptosis.^46^ TH results in a prolonged neonatal intensive care unit (NICU) stay, separation from mother, delayed breastfeeding initiation, risks associated with sedative medications, risk of coagulopathy and pulmonary hypertension and ultimately increased health care economic costs.^6,47^ Sedation is often required in infants undergoing TH due to the discomfort associated with a low core temperature. Concerns have been raised about commonly used drugs such as morphine as it may contribute to neuronal and microglial apoptosis. Tolerance has also been described requiring increased doses and problems with withdrawal on discontinuation.^48–50^

Therefore, it seems logical that a randomised trial of TH in infants with mild HIE is now required.^51^ However, consensus must be reached on how we identify these infants. Improved identification and selection of infants who may potentially benefit from TH would limit the numbers required to power such a study.

Our current methods of identifying infants are flawed as the primary and most widely available assessment is based on clinical examination, which is highly subjective.^52^ Although several studies have assessed different scoring tools,^3,4,53,54^ it is often difficult to clinically differentiate between mild and moderate grades of HIE.^10,11^ Furthermore, it is based on a modified Sarnat score, which was initially validated to examine infants repeatedly and at 24 hours.^2^ Many centres now use the Thompson score. Initially developed as a quick and easy tool to assess infants with encephalopathy, it was also developed to examine infants on a daily basis and is most predictive of outcome on days 3–4.^3^ Early EEG has been shown to be superior to clinical examination alone for the prediction of outcome.^31,55^

aEEG is preferred in many neonatal units as it can be easily applied and interpreted through pattern recognition. In the pre-TH era, aEEG within 6 hours of birth was the best predictor of outcome and aEEG was incorporated into many of the TH randomised controlled trials as an inclusion criteria for randomisation. As mentioned, different EEG or aEEG machines use slightly different algorithms to generate an aEEG channel. For quantitative analysis, we used rEEG, which has a standard definition.^38^ This allowed us to assess the ability of aEEG to distinguish between infants with mild HIE and those without HIE in our study. In our cohort, rEEG features alone such as median amplitude, upper and lower margins did not distinguish infants with mild HIE from the non-HIE group. Studies have previously shown a discrepancy in HIE grade between continuous multichannel EEG and aEEG in the same infants.^56^ This is likely due to the fact that the raw EEG is uncompressed and specific features such as sharp and slow waves, short periods of discontinuity, asymmetry and asynchrony can be easily seen but these are lost in the compressed or summarised aEEG. Nonetheless, aEEG is very useful for the visual identification of SWC, a feature that was absent or poorly defined in our cohort of infants with mild HIE. While continuous multichannel EEG may not be available in all the neonatal units, most aEEG devices allow visualisation of at least some raw EEG channels that can provide richer information and a more enhanced aEEG interpretation.

Multichannel EEG does require expert interpretation and many units do not have 24 hour access to a neonatal neurophysiologist or neurologist. Quantitative EEG analysis of multichannel EEG provides an automated objective description of the EEG without the need for expert interpretation. It also has the potential to detect more subtle differences, which may not be easily identified on visual assessment alone. In this cohort, quantitative analysis demonstrated significant differences in all measures of spectral shape at the lower frequency bands when compared to the non-HIE group. These features of the EEG would be very difficult, if not impossible, to detect visually. Quantitative EEG features provides a scalable, continuous and objective assessment of the EEG that may be useful in the future to improve our identification of at-risk infants. It is also easily applied to a smaller number of EEG channels.

Our study is limited by the fact that it was a retrospective analysis of data; however, EEGs of infants with HIE were collected over different cohorts in both the TH and pre-TH era capturing the clinical variability in presentation. In addition, it was not possible to completely blind the neurophysiologists conducting the qualitative analysis to study group; however, their findings were remarkably consistent with the objective quantitative analysis. Outcome data is currently unavailable for the entire population; however, neurodevelopmental follow-up is underway. We plan to clinically follow these infants to 5 years of age to determine which, if any, of these abnormal features correlate with outcome. We do know from previous studies that a proportion of infants with mild HIE will have cognitive or behavioural disability on follow-up; however, these difficulties may not be evident until 5 years of age or later.^15^

Mild HIE is not “normal” as previously thought^2,12,13^; these infants have an encephalopathy and may have significant learning, behavioural and emotional difficulties on follow-up yet have largely been ignored by research to date.^15,16,18^ Our current criteria of identifying infants at risk of long-term developmental issues is clearly inadequate as many infants who have significant disability on follow-up currently fall outside the current criteria for intervention.

In conclusion, this is the first study to describe early multichannel EEG findings (<6 hours of age) in infants with mild HIE and compare them with healthy term infants at the same time point. There are clear differences between the EEGs of infants with mild HIE and healthy term infants. Visual analysis shows that 72% of infants with mild HIE have some abnormal EEG features such as sleep cycle disruption or excessive sharp and slow waves within 6 hours of birth, which cannot all be attributed to the difficult sleep environment of the NICU. Quantitative analysis of the EEG reveals significant differences in spectral measures of the lower frequency bands. The challenge now is to correlate these features with outcome and determine the importance of each feature. Incorporation of early quantitative EEG features could be useful for future trials of TH in infants with mild HIE to aid in the early and objective identification of cases.

## Supplementary information

Supplementary Table 1
