# Supplementary material for: Multichannel EEG abnormalities during the first 6 hours in infants with mild hypoxic–ischaemic encephalopathy
Source: Pediatr Res. 2021 Apr 20;90(1):117–24. doi: 10.1038/s41390-021-01412-x (PMC8370873; doi:10.1038/s41390-021-01412-x)
Supplement: Supplementary file 1 — Supplementary Table 1 [file 41390_2021_1412_MOESM1_ESM.pdf]

Supplementary Table 1. Modified Sarnat Exam

Stage	Normal	Mild	Moderate	Severe
**1. Level of Consciousness**	Normal	Hyper-alert/Irritable	Lethargic/Obtunded	Stupor/Coma
**2. Spontaneous Activity**	Normal	Normal	Decreased	Absent
**3. Muscle Tone**	Normal	Normal	Mild Hypotonia	Flaccid
**4. Posture**	Normal	Mild Distal Flexion	Strong Distal Flexion	Decerebrate
**5. Primitive Reflexes**				
Suck	Normal	Weak	Weak/Absent	Absent
Moro	Normal	Strong/Low Threshold	Weak/Incomplete/ High Threshold	Absent
**6. Autonomic Function**				
Pupils	Normal	Mydriasis	Miosis	Unequal/Fixed/ Dilated/Poor Reflex
Heart Rate	Normal	Tachycardia	Bradycardia	Variable
Respirations	Normal	Normal	Periodic Breathing	Apnea

For Review Only
